# Cortisol as a Mediator of Prenatal Distress and Difficult Infant Temperament: A Systematic Review and Meta‐Analysis Protocol

**DOI:** 10.1002/hsr2.71309

**Published:** 2025-10-24

**Authors:** Ferdinand Sörensen, Emma Fransson, Alkistis Skalkidou, Ingeborg Krägeloh‐Mann, Birgit Derntl

**Affiliations:** ^1^ Pediatric Neurology & Developmental Medicine University Children's Hospital Tübingen Tübingen Germany; ^2^ Department of Psychiatry and Psychotherapy, Tübingen Center for Mental Health (TüCMH) University of Tübingen Tübingen Germany; ^3^ Department of Women's and Children's Health Uppsala University Uppsala Sweden; ^4^ German Center for Mental Health (DZGP) Tübingen Germany

**Keywords:** cortisol, fetal programming, glucocorticoid system, infant temperament, meta‐analysis, prenatal distress, review

## Abstract

**Background and Aims:**

Exposure to prenatal distress is associated with a risk of developing difficult temperament in infants, an indicator for a higher likelihood of later adverse developmental outcomes, including behavioral and mental health problems. The underlying biological mechanisms of this association are still unclear, but many support the idea of fetal programming, which postulates the influence of environmental factors on the fetus during pregnancy. Evidence points to the role of cortisol as a mediator of stress, but the results are inconsistent. In this systematic review and meta‐analysis, the association between prenatal distress and difficult infant temperament will be assessed, focusing on cortisol exposure as a possible mediator and including subgroup analyses.

**Methods:**

Literature research will be performed in PubMed, Web of Science, MEDLINE, PSYNDEX, APA PsycArticles, APA PsycInfo, and CINAHL. Inclusion criteria are the availability of (self‐) assessment of maternal prenatal distress, maternal prenatal cortisol levels, and (parental) assessment of difficult infant temperament up to the age of 2 years. Screening and selection of peer‐reviewed English (or German) articles and assessment of article quality will be done by two independent reviewers, with a third one included in the case of disagreement. Effect sizes will be extracted and subgroup analyses will be performed not only for covariables but also for the methods of assessing prenatal distress, cortisol, and temperament. This protocol follows the PRISMA‐P checklist.

**Conclusion:**

This systematic review and meta‐analysis will contribute to the ongoing discussion of whether and how cortisol mediates the association between prenatal maternal distress and difficult infant temperament. Identifying sensitive subgroups is an integral part of this study, as results might guide further research to vulnerable population groups.

**Abbreviation**
PD: prenatal distress
11β‐HSD2: 11β‐hydroxysteroid dehydrogenase type 2

## 1 Background

The fetal programming hypothesis states that the early prenatal environment influences the development of the fetus, first reported in humans [1, 2] and subsequently explored in animal models [3]. Examples of this were first observed for nutritional factors [1, 4, 5] and later for addictive substances, including alcohol [6], nicotine [7], and various drugs [8]. In recent years, the impact of psychological factors on fetal programming, such as maternal prenatal distress (PD), has also been investigated with glucocorticoid hormones as possible mediators. The notion of PD contains a wide variety of factors, including severe distress, such as natural disasters [9, 10], and chronic distress, such as individuals diagnosed with depression and anxiety [11, 12]. The effects of prenatal maternal distress might be mediated via the maternal glucocorticoid system, which is often mentioned as a possible biological mechanism of fetal programming. Most research focuses on cortisol as the most prominent biomarker of stress [13]. It is released by the adrenal glands within the hypothalamus–pituitary–adrenal (HPA) axis system and involved in a variety of metabolic pathways to mobilize energy. The exact biological mechanisms of the association between maternal PD and adverse offspring outcomes, such as difficult temperament and later impaired neurodevelopment [14], remain to be elucidated. It is speculated that this association is mediated via the placental enzyme 11β‐hydroxysteroid dehydrogenase type 2 (11β‐HSD2) [15]. This enzyme inactivates cortisol into the biologically inactive cortisone. Evidence suggests that the ability to upregulate 11β‐HSD2 expression is diminished during maternal chronic stress [16, 17, 18] and may also be influenced by interindividual variability in 11β‐HSD2 [19, 20]. This could cause higher levels of maternal cortisol to enter the fetal bloodstream which might lead to changes in the glucocorticoid regulation, for example, (epigenetic) programming of the HPA axis and neurodevelopment [21, 22, 23], potentially reducing the number of glucocorticoid receptors, thereby affecting the negative feedback loop of glucocorticoid hormones [24, 25].

Accordingly, several studies observed a positive association between PD and maternal cortisol levels [17], for review, see Glover [26] and Kataja et al. [27]. However, other studies have reported no such association [28, 29, 30, 31, 32, 33, 34, 35]; for review, see Zijlmans et al. [36].

The association between prenatal maternal cortisol levels and offspring outcome, such as impaired neurodevelopment, delayed cognitive and motor development, impaired behavioral response to stressful situations, has been consistently reported in animal models (for review see Seckl and Meaney [19]; Fatima et al. [37]; and Pallarés and Antonelli [38]). In humans, several studies indicated that maternal cortisol mediated the effects of PD on the offspring, including reports of higher internalized symptoms in girls of mothers with higher depression scores [17] or atypical emotional processing observed in infants of mothers exposed to synthetic glucocorticoids [27]. The effects of PD also include adverse birth outcomes such as reduced birth weight and infant developmental trajectories, as shown by several animal models (for review see Bohacek and Mansuy [39]; Rakers et al. [40]; Weinstock [41]) and human data (for review see Glover et al. [15]; Rakers et al. [40]; Berretta et al. [42]; Stein et al. [43]).

Research in areas like nutrition highlights the role of the first 1000 days as a time of vulnerability [44], including the prenatal period and the first 2 years postpartum. The detrimental effects of PD could lead to a mismatch between the “programmed” intrauterine and extrauterine environment [45] that results in adverse infant outcomes, including elevated difficult infant temperament [46].

Difficult infant temperament is defined by Thomas et al. [47] as negative mood, withdrawal, low adaptability, high intensity, and low regularity. It is often assessed via parental questionnaires or via experimental ratings [48] and is measurable as early as 2 days after birth [49]. It has been discussed as an indicator of later behavioral [50] and neuropsychiatric problems [14] and as a critical factor for developmental trajectories [51]. Associations between infant temperament and later psychopathology like psychotic disorders have been shown to remain until early adulthood [52, 53]. How PD influences difficult infant temperament is not clear, but underpinnings of temperament comprise biological reactions including the HPA axis [54].

Few studies have assessed PD in combination with maternal cortisol as a mediator and difficult infant temperament as the outcome. In a systematic review, prenatal depression and anxiety and maternal cortisol were identified as being associated with infant temperament [55]. Moreover, maternal anxiety/depression and maternal cortisol during pregnancy predicted higher infant negative reactivity [56], and increased difficult temperament and lower fine motor development at age 16 months [57]. Sex dependent effects have also been observed, as maternal prenatal cortisol predicted higher negative emotionality in female infants while male infants showed less negative emotionality [58]. In contrast, other studies do not find an association between PD, maternal depression, and infant temperament [59, 60, 61]. O'Donnell and Meaney [62] suggest, due to the inconsistency of results in the literature, that the association between maternal mental health and cortisol might be only assessable in subgroups of the population, such as individuals with more severe mental health conditions.

Taken together, previous results on the association between maternal PD, cortisol as mediator and difficult infant temperament remain inconsistent [36]. It is still debated whether prenatal maternal cortisol acts as a general mediator for maternal PD or if its effects are specific to subgroups. These subgroups could include, for instance, mothers with varying activity levels of 11ß‐HSD2 enzyme possibly due to chronic distress exposure [18, 19, 20, 63] or a specific sex [64]. This systematic review and meta‐analysis investigate the associations between maternal prenatal cortisol as a mediator between PD and difficult temperament in infants during the first 2 years of life.

Specifically, we hypothesize that (1) maternal prenatal PD is associated with difficult infant temperament in infants during the first 2 years of life, (2) PD is associated with maternal prenatal cortisol levels, and (3) maternal prenatal cortisol is associated with difficult infant temperament. Given previous mixed findings, we will also investigate whether this mediation effect differs by type of PD, for instance, chronic maternal distress (e.g., prenatal anxiety, prenatal depression, perceived stress) versus acute stressors (e.g., exposure to disasters, trauma, or sudden life events) [65]. Since prenatal and postpartum distress, such as maternal depression and anxiety, are highly comorbid [65], we will assess whether postpartum distress moderates or confounds these associations. Additionally, to account for heterogeneity across studies, we will conduct subgroup analyses based on: study design, such as experimental and observational studies, as methodological differences may introduce a bias [66]; assessment tools for PD and infant temperament, for example, self‐report measures may lead to different results compared to observation methods or clinical diagnosis [48]; infant sex due to evidence of sex‐specific programming effects [17, 67]; cortisol sample type such as saliva, blood, hair, and urine [68]; timing of cortisol collection, early versus late pregnancy, as the effect of cortisol in early pregnancy may be less pronounced as in later stages [69, 70] and study quality to assess potential bias in findings. This study follows PRISMA‐P guidelines [71] and will be registered in PROSPERO, an international database for systematic reviews.

By synthesizing the literature, this systematic review and meta‐analysis aims to provide insights into the associations between PD and difficult infant temperament with maternal prenatal cortisol as mediator and whether its role varies across subgroups, contributing to a better understanding of fetal programming mechanisms.

## 2 Methods

### 2.1 Information Sources

To identify relevant studies, a systematic literature search will be conducted in multiple electronic databases, including PubMed, Web of Science, MEDLINE, PSYNDEX, APA PsycArticles, APA PsycInfo, and CINAHL. Google Scholar will be used as a supplementary resource to identify potential additional references. However, it will not be included in the main systematic search strategy, as its AI‐driven search algorithms make results nonreproducible.

The search strategy will incorporate Boolean operators and wildcards to enhance the comprehensiveness of the search. In PubMed, MeSH terms (Medical Subject Headings) and the [tiab] (title and abstract) field code will also be utilized.

### 2.2 Search Strategy

A librarian from the University Library of Tübingen was included as an advisor for the search strategy.

PubMed (with MeSH terms):

(“MOTHERS”[MESH] OR “MATERNAL”[TIAB]) AND (“Pregnancy”[Mesh] OR prenatal OR antenatal) AND (“HYDROCORTISONE” [MESH] OR GLUCOCORTICOI* OR CORTISOL) AND (“INFANT”[MESH] OR INFANT*[TIAB] OR TODDLER*[TIAB] OR NEWBORN [TIAB]) AND (“TEMPERAMENT” OR “AFFECTIVITY” OR EMOT* OR REGUL*).

Other databases (without MeSH terms):

(mothers OR maternal) AND (pregnancy OR prenatal OR antenatal) AND (hormones OR hydrocortisone OR glucocorticoi* OR cortisol) AND (infant OR infant* OR toddler* OR newborn) AND (temperament OR affectivity OR emot* OR regul*).

### 2.3 Study Screening and Selection

Titles/abstracts of the retrieved studies will be screened by two independent reviewers to identify relevant studies. In a second step, the full texts of the studies will be retrieved and the Abstracts and Methods sections will be assessed. If all inclusion criteria are met, the study will be included in the systematic review and, depending on the availability of the data, also in the meta‐analysis. If the results of the reviewer's assessments differ, a third reviewer will be consulted to reach a consensus. Articles will be imported into Zotero [72] as the preferred reference management software, to facilitate easy recognition of duplicates and handling of retrieved articles.

### 2.4 Data Extraction and Collection

A standardized data extraction form will be used, and a pilot test will be conducted to assess the form's quality and to include any additional data extraction criteria. The form will include (preliminary): author, year, study design, sample categorization (general population/clinical sample), location of the study, sample size, assessment methods of PD, and timepoints for maternal PD, sample type, such as, blood, saliva, urine, hair, and trimester of cortisol collection. To characterize the mothers, the following information will be additionally collected if available: maternal age, parity, complications during childbirth, maternal mental or physical health (problems), postpartum distress, twin pregnancy; socioeconomic factors like: employment status, relationship status, experience with interpersonal violence. If available, similar information will be gathered on the fathers. For infants: female/male ratio of offspring, birth weight, gestational age, APGAR scores (5 and/or 10 min), infant temperament assessment tools and, age of infants when assessed.

**Figure 1:**
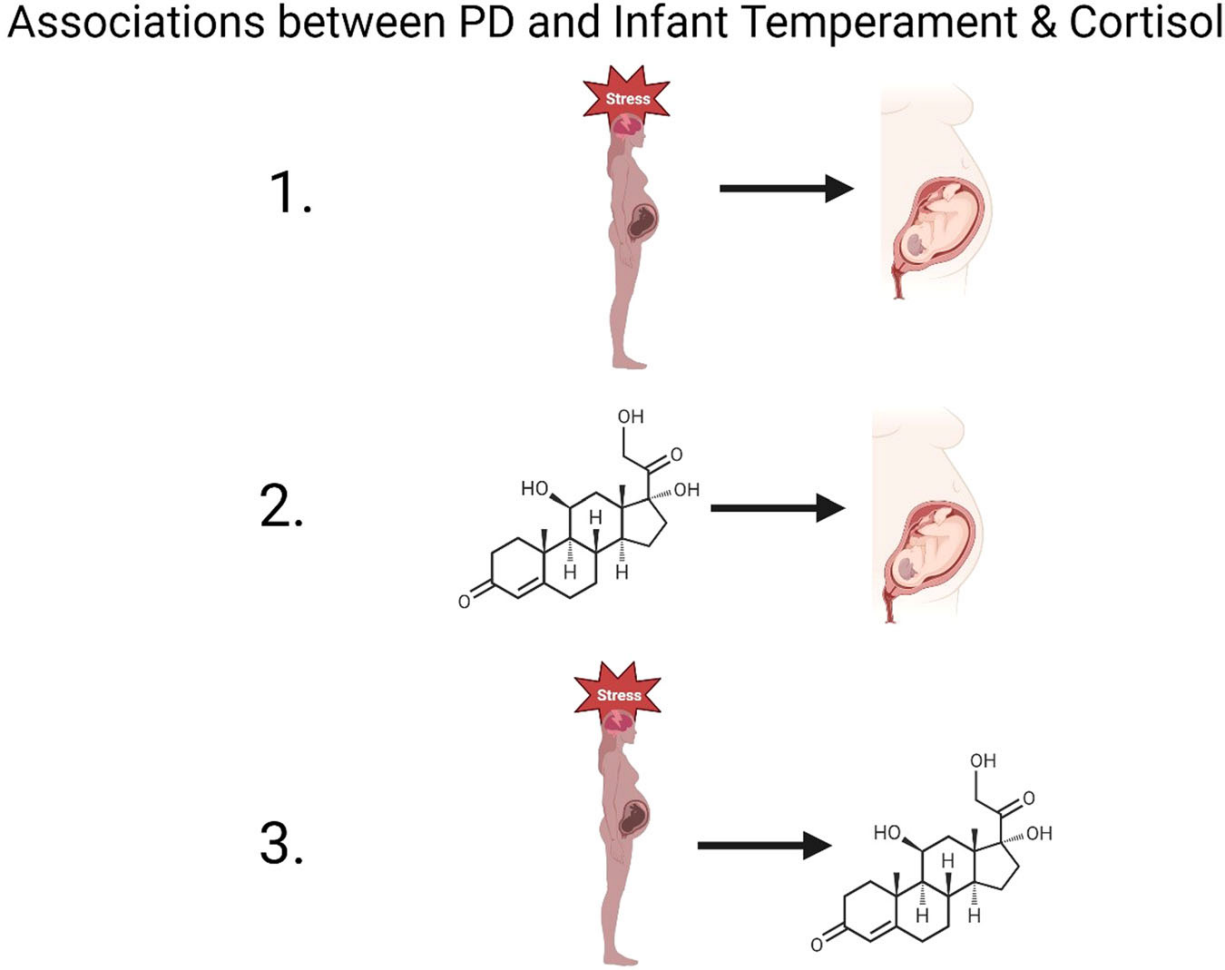
Overview of associations to be examined in the review. (1) PD and difficult infant temperament, (2) maternal prenatal cortisol and difficult infant temperament, and (3) PD and maternal prenatal cortisol.

Association size measures will be collected for the association between (1) maternal PD and difficult infant temperament in infants during the first 2 years of life; (2) maternal PD with maternal prenatal cortisol levels; (3) maternal prenatal cortisol levels and difficult infant temperament (Figure 1). As mostly cohort studies with continuous outcome measures are expected to be included, correlation coefficients and sample sizes will be primarily extracted as measures of effect size for every association.

### 2.5 Eligibility Criteria

Articles are available in English or German and have been published in a peer‐reviewed journal, without timeframe restrictions. Certain formats of text will be excluded, including books, protocols, position and opinion papers, editorial letters, intervention studies, unpublished data, reviews, and meta‐analyses. The systematic review and the meta‐analysis will include studies reporting maternal PD, cortisol levels during pregnancy, and difficult infant temperament outcomes up to the age of 2 years.

Most studies are expected to assess PD and temperament via self‐reported questionnaire data, studies with experimental designs will also be included and analyzed as a subgroup. The anticipated study designs include experimental and observational (prospective and longitudinal observational, cohort and case‐control) studies to ensure causal inference. Cross‐sectional studies which include only one timepoint will be excluded.

The target population includes pregnant women and their infants. The studies may encompass diverse populations from various geographical locations. The inclusion criteria for the study population will not be restricted by factors such as maternal age, parity, complications during childbirth, or socioeconomic status.

### 2.6 Quality of Evidence and Risk of Bias

The quality of the included studies will be evaluated by two independent reviewers using the Newcastle–Ottawa Scale [73], a widely used tool to assess the quality of studies. In cases where discrepancies arise between the reviewers, a third reviewer will be involved to reach a consensus. Studies of low quality, as determined by the assessment, will be excluded from the final analysis to ensure the reliability of the findings. The risk of bias within studies will be evaluated using statistical methods such as the Egger test and funnel plots. In addition, regression tests for funnel plot asymmetry will be conducted. Study heterogeneity will be assessed using Cochran's *Q* statistic, *I* ^2^ (inconsistency) statistic, and *τ* ^2^ (tau‐squared) statistic. The identification of influential studies will be done using various measures, including externally standardized residuals, DFFITS values, Cook distances, covariance ratios, leave‐one‐out estimates, hat values, and weights.

### 2.7 Statistical Analysis

A first descriptive analysis will be conducted, to identify commonly used instruments and methodologies to assess PD, cortisol, and difficult infant temperament. Pooling of the effect sizes will be performed using standardized Fisher‐*Z* scores for correlational effect sizes in a random effects model. The heterogeneity of included studies will be calculated. Sources of heterogeneity will be assessed via sensitivity and subgroup analysis. The subgroups will include type of PD, specifically chronic maternal distress (e.g., prenatal anxiety, prenatal depression, perceived stress) and acute stressors (e.g., exposure to disasters, trauma, or sudden life events); study design (experimental vs. observational); assessment methods for both PD and infant temperament (e.g., self‐report questionnaires, observational measures, clinical diagnoses); infant sex; types of cortisol samples (e.g., saliva, blood, hair, urine); timing of cortisol collection, categorized by trimester rather than analyzed continuously, as trimester‐based analysis allows for standardized comparisons across studies; presence of postpartum distress as a potential moderator or confounder; and overall study quality to assess potential biases. These analyses depend on the number and quality of studies found and will be conducted if the number of available studies is sufficient (≥ 2). Based on previous reviews [36], the number of retrieved articles is anticipated to be small, it is not conceivable whether all planned subgroup analyses will be feasible. The analysis will be conducted using R, including the *meta* [74] and *metafor* package [75]. Besides the quantitative evaluation, a systematic review and a summary of all included studies will be created if a quantitative synthesis is not appropriate.

**Figure 2:**
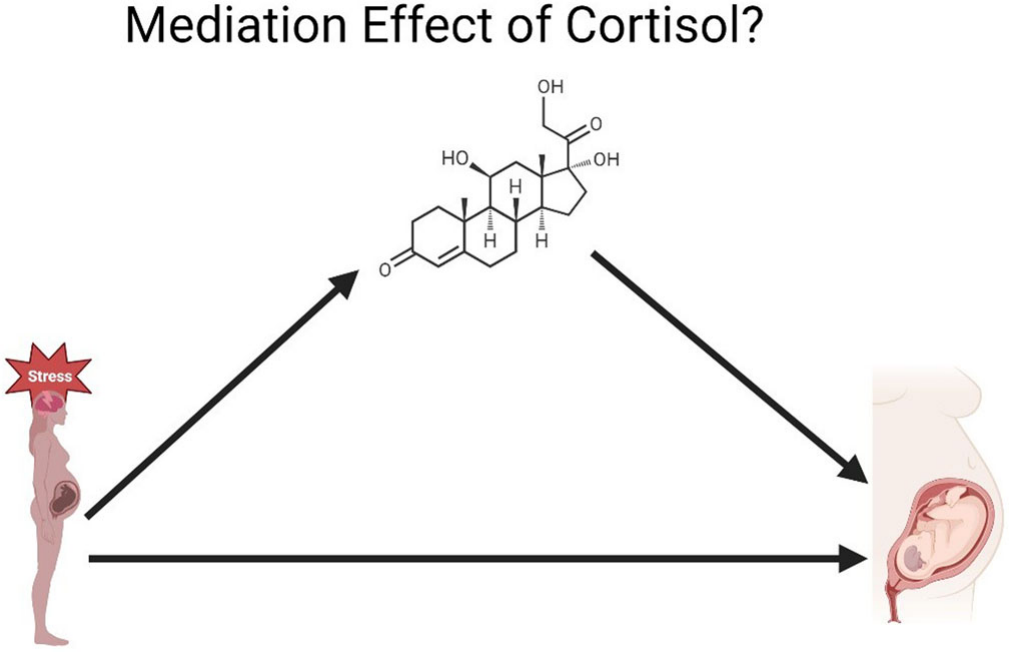
Mediation model: maternal prenatal cortisol as a mediator between prenatal maternal distress and infant difficult temperament.

Forest plots to assess the observed effect of the selected studies will be computed. We will calculate the following associations: (1) maternal prenatal PD and difficult infant temperament; (2) maternal PD and maternal prenatal cortisol levels; (3) maternal prenatal cortisol level and difficult infant temperament (Figure 1). Additionally, we will evaluate the mediation effect of maternal cortisol (Figure 2), including all studies in which maternal cortisol was measured after PD exposure, and perform meta‐regressions to estimate the influence of maternal cortisol levels while accounting for postpartum distress and potential confounding factors. However, studies in which PD is conceptualized as a chronic condition (e.g., maternal depression or anxiety during pregnancy) will also be included, even if PD and maternal cortisol were assessed simultaneously, to account for the chronic nature of maternal depression and anxiety.

## 3 Results

### 3.1 Data Reporting

A systematic review summarizing the results of the retrieved studies will be conducted, providing a comprehensive overview of the findings. If the quality and homogeneity of the included studies allow for a quantitative synthesis, a meta‐analysis will be performed, incorporating the planned subgroup and a mediation analysis. The data reporting will follow the recommendations by Assel et al. [76]. The systematic review and meta‐analysis will adhere to the Preferred Reporting Items for Systematic Reviews and Meta‐Analyses (PRISMA) guidelines [77].

## 4 Discussion

In conclusion, this systematic review and meta‐analysis aims to provide a thorough and evidence‐based understanding of the association of maternal PD and difficult infant temperament, with a focus on the potential mediating role of cortisol exposure. The inclusion of subgroup analyses will allow for a nuanced examination of the associations in different contexts. By following a rigorous methodology and adhering to established reporting guidelines, this meta‐analysis will contribute valuable insights to the existing literature, inform clinical practice, and guide future research in this field.

## Author Contributions

**Ferdinand Sörensen:** conceptualization, investigation, methodology, visualization, writing – review and editing, writing – original draft, software, formal analysis, project administration, data curation, validation. **Emma Fransson:** writing – review and editing. **Alkistis Skalkidou:** writing – review and editing. **Ingeborg Krägeloh‐Mann:** writing – review and editing. **Birgit Derntl:** writing – review and editing, funding acquisition, supervision, validation, resources. All authors reviewed the paper. All authors read and approved the final manuscript.

## Ethics Statement

The authors have nothing to report.

## Consent

The authors have nothing to report.

## Conflicts of Interest

The authors declare no conflicts of interest.

## Transparency Statement

The lead author Ferdinand Sörensen affirms that this manuscript is an honest, accurate, and transparent account of the study being reported; that no important aspects of the study have been omitted; and that any discrepancies from the study as planned (and, if relevant, registered) have been explained.

## Data Availability

The data sets analyzed during the current study will be available from the corresponding author upon reasonable request.
